# A Machine Learning Approach to Predicting Mortality Risk in Chemotherapy-Treated Lung Cancer: Machine Learning Model Development and Validation

**DOI:** 10.2196/72424

**Published:** 2025-12-18

**Authors:** Jianjun Zou, Jinyi Huang, Katie Lu, Ao Lin, Chen Xie, Jinrong Zhang, Boqi Rao, Zhi Li, Dongming Xie, Ling Lu, Feng Luo, Jinbin Chen, Lei Yang, Fuman Qiu, Xin Zhang, Yibin Deng, Jiachun Lu

**Affiliations:** 1 Institute of Tuberculosis Guangzhou Medical University Guangzhou, Guangdong China; 2 Guangzhou Key Laboratory of Tuberculosis Research Guangzhou Chest Hospital Guangzhou, Guangdong China; 3 Department of General Medicine Guangzhou Chest Hospital Guangzhou, Guangdong China; 4 School of Public Health Guangzhou Medical University Guangzhou, Guangdong China; 5 School of Medicine University of Arizona Tucson, AZ United States; 6 State Key Laboratory of Respiratory Disease Guangzhou Medical University Guangzhou, Guangdong China; 7 Department of Thoracic Surgery The First Affiliated Hospital of Guangzhou Medical University Guangzhou, Guangdong China; 8 Emergency Department The Second Affiliated Hospital of Guangzhou Medical University Guangzhou China; 9 KingMed School of Laboratory Medicine Guangzhou Medical University Guangzhou China; 10 The Key Laboratory of Advanced Interdisciplinary Studies The First Affiliated Hospital of Guangzhou Medical University Guangzhou, Guangdong China; 11 The Institute for Chemical Carcinogenesis Guangzhou Medical University Guangzhou, Guangdong China; 12 Guangzhou Institute of Respiratory Health Guangzhou Medical University Guangzhou China; 13 Centre for Medical Laboratory Science The Affiliated Hospital of Youjiang Medical University for Nationalities Baise China

**Keywords:** lung cancer, chemotherapy, mortality, machine learning, prediction model

## Abstract

**Background:**

Accurately predicting the survival outcomes of patients with lung cancer receiving chemotherapy remains challenging.

**Objective:**

To improve clinical management of this population, this study developed a multivariate machine learning (ML) model to assess all-cause mortality risk in chemotherapy-treated patients with lung cancer.

**Methods:**

This study retrospectively recruited 1278 postchemotherapy patients with lung cancer from Guangzhou Chest Hospital between 2017 and 2019. Candidate features such as demographic characteristics, environmental exposures, clinical information, and patient-reported symptoms were collected via questionnaires and the electronic medical record system. The survival status and the deceased date were investigated twice a year. A total of 84 predictive models were constructed on the training set using 5 ML algorithms either individually or in pairwise combinations. The concordance index was used to identify the optimal model on the testing set, with performance validated via receiver operating characteristic curves, calibration curves, and decision curve analysis. Additionally, Shapley Additive Explanations and restricted cubic splines were applied for feature attribution analysis.

**Results:**

The optimal model ultimately retained 21 prognosis-association features, including age, sex, BMI, smoking status, environmental smoke, the MD Anderson Symptom Inventory for Lung Cancer total score trajectories, cluster of differentiation 56, TNM stage, histology, and prechemotherapy blood biomarkers. On the testing set, the model acquired a concordance index of 0.702 (95% CI 0.652-0.753). The decision curves demonstrated positive clinical benefit when the risk thresholds were 0.40-0.69, 0.62-0.99, and 0.72-0.99 for 1-, 3-, and 5-year mortality predictions, respectively. The calibration curves showed that the predicted mortality probabilities fluctuated around the observed probabilities, and the Brier scores for 1-, 3-, and 5-year predictions were 0.20, 0.18, and 0.11, respectively. The area under the curve of the model was 0.740, 0.777, and 0.915 for 1-, 3-, and 5-year mortality predictions, respectively. Interpretability feature attribution analysis revealed that the significant features could predict all-cause mortality risk in chemotherapy-treated patients with lung cancer.

**Conclusions:**

Our ML models exhibited acceptable discrimination, calibration, and clinical benefit in predicting the mortality risk of chemotherapy-treated patients with lung cancer, which could help clinicians in personalized prognostic management.

## Introduction

Lung cancer is the leading cause of cancer-related deaths globally. Lung cancer claimed approximately 1.8 million lives in 2022 alone, accounting for 18.7% of global cancer deaths [1]. With the advancements in medicine, the emergence of targeted and immune drugs introduces new options for lung cancer treatment. Personalized treatment strategies combining surgery, radiotherapy, chemotherapy, targeted therapy, and immunotherapy demonstrate significant efficacy in improving the living quality and long-term survival rates of patients with lung cancer. However, for some advanced lung cancers lacking driver mutations and with negative immune checkpoint expression, chemotherapy remains the primary treatment modality [2,3]. Thus, accurately predicting the mortality risk of chemotherapy-treated lung cancers to optimize treatment planning and patient care is of significant public health importance.

Machine learning (ML) models have become increasingly important tools for predicting the mortality risk of patients with lung cancer treated with chemotherapy, offering new insights and improved accuracy over traditional methods. While demographic and clinical characteristics are important, recent research has highlighted the potential benefits of incorporating additional data sources to enhance model performance [4-6]. For example, although the TNM stage is a recognized factor associated with the prognosis, it explains just less than 30% of the prognostic differences [7,8]. It is noted that genomic biomarkers present a means of refining prognosis assessment models, but the expensive genetic testing restricts the clinical application [9-11]. The above situation underscores the requirement for a more effective and interpretable mortality risk stratification model for postchemotherapy patients with lung cancer.

The biopsychosocial framework advocates dissecting disease from diverse angles, including symptom burden. The MD Anderson Symptom Inventory for Lung Cancer (MDASI-LC), a patient-reported outcome (PRO) tool, offers key perspectives into the self-reported symptom experience of patients receiving chemotherapy [12]. Integrating longitudinal PRO information into prognostic models has notable value in refining the prediction of patient outcomes [13-16].

This study adheres to the transparent reporting of a multivariable prediction model for individual prognosis or diagnosis guidelines as much as possible to ensure the transparent and accurate reporting of our predictive model development and validation [17]. This study developed a multivariable ML model that integrated demographic characteristics, clinical features, environmental factor exposure, immunohistochemical indicators, and total PRO score to predict the mortality risk of chemotherapy-treated patients with lung cancer. The model aims to provide clinicians with an effective tool for predicting the risk of death in patients with lung cancer after chemotherapy, assisting clinicians in customizing treatment plans, optimizing patient care plans, and improving patient prognosis.

## Methods

### Overview

**Figure 1:**
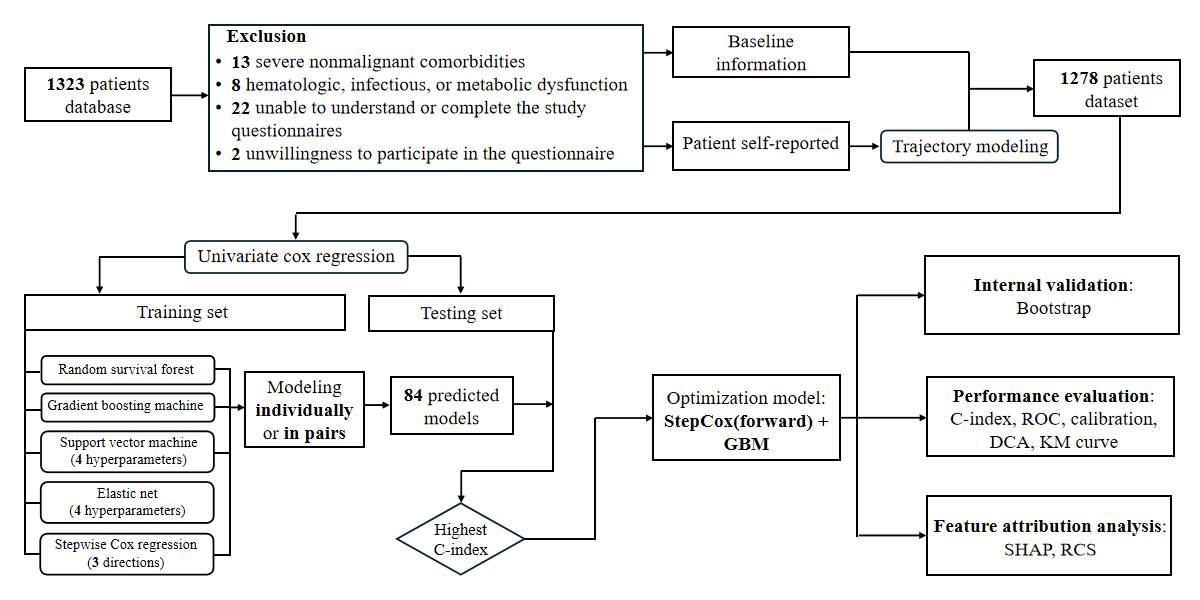
The flowchart of this study. This study provided a comprehensive framework for building machine learning models to predict mortality risk for patients with lung cancer after chemotherapy. C-index: concordance index; DCA: decision curve analysis; GBM: gradient boosting machine; KM: Kaplan-Meier; RCS: restricted cubic spline; ROC: receiver operating characteristic curve; SHAP: Shapley Additive Explanations.

An overview of the entire study design and ML framework is provided in Figure 1.

### Study Population

This retrospective and semilongitudinal investigation included 1278 patients with lung cancer who received platinum-based chemotherapy at Guangzhou Chest Hospital between November 2017 and 2019. Demographic characteristics, environmental exposures, clinical information, and patient-reported symptoms were collected using questionnaires and the electronic medical record system. Patients’ TNM stage was identified by the seventh edition of the classification system.

Patients who met the following inclusion criteria were included (1) histologically confirmed diagnosis of primary lung cancer, (2) absence of prior chemotherapy for lung carcinoma, and (3) demonstrated ability to comprehend and complete study questionnaires with clear consciousness.

Patients with the following presentations that would bias their symptom reporting or outcome were excluded: (1) severe nonmalignant comorbidities; (2) hematopoietic, infectious, or metabolic dysfunction; and (3) preexisting psychological disorders. The specific number of excluded participants is presented in the flowchart of Figure 1.

### Patient-Reported Symptom

Patients completed self-report questionnaires at admission and the first 4 chemotherapy cycles. The professional questionnaires included (1) the MDASI-LC: this is a validated PRO tool specifically designed to assess symptom severity in patients with lung cancer. It consists of 22 items divided into 3 parts: core symptoms, lung cancer-specific symptoms, and additional symptoms. Each item is scored on a scale from 0 to 10, with higher scores indicating more severe symptoms [18]. (2) The Karnofsky Performance Status (KPS): this is a widely used tool for assessing the overall health status and functional state of patients. The KPS score ranges from 0 to 100, with higher scores indicating better functional status. It is commonly used in clinical practice to evaluate patient prognosis and treatment response, and it has been validated in various studies [19]. (3) The Zubrod Performance Score (ZPS): this tool assesses the physical condition of patients, with scores ranging from 0 to 5. Higher scores indicate worse physical status. ZPS is another widely used tool in clinical practice, providing valuable insights into patients’ daily living abilities and overall health [20].

### The Primary End Point

All-cause mortality served as the primary end point in this study. The professionals monitored the patients biannually by telephone. The death date was recorded if the patients had died; otherwise, they would be followed until death. The time horizon for this outcome is defined as 1-, 3-, and 5-year posttreatment initiation. The follow-up period in this research ranged from 1 month to 11 years.

### Statistical Analysis Methods

The normality of continuous variables was assessed using the Shapiro-Wilk test, and the group comparisons were made using the 2-tailed *t* test or the Mann-Whitney *U* test. Categorical variables were analyzed using the chi-square or the Fisher exact test. Survival curves were generated using Kaplan-Meier and compared using the log-rank test. Hazard ratio and 95% CI were estimated using Cox regression. Statistical significance was defined when *P*<.05 (2-tailed). Missing data imputation was performed using the missForest algorithm, which accommodated mixed data types and handled nonlinearities and outliers.

The reliability of the scales was assessed using Cronbach α to measure internal consistency. Pearson correlation coefficients were calculated to examine the correlations between the 3 scales, assessing their validity. Longitudinal symptom burden trajectories were identified using group-based trajectory modeling (GBTM), with data from the 3 scales assessed across 5 separate time points [21]. GBTM criteria were odds of correct classification >5, average posterior probability (Avepp) >0.7, posterior probability of group membership (*P*_j_) >5%, correspondence between *P*_j_ and probability of group membership (π_j_), relative entropy (*E*_k_) >0.7, and a minimized Bayesian information criterion value.

### ML Methods

Random survival forest (RSF), gradient boosting machine (GBM), survival support vector machine (survival-SVM), elastic net (Enet), and stepwise Cox regression were used to develop a prognostic model [22-25]. For the RSF and GBM models, the best hyperparameters for these 2 models were determined by constructing separate base models on the training set and performing preliminary cross-validation and performance evaluation. For the hyperparameter γ of the survival-SVM model, the hyperparameter α of the Enet model, and the 3 directions of the stepwise Cox regression model, a grid search method was used. The specific parameters of the five algorithms are as follows: (1) RSF: ntree=1000, nodesize=32, mtry=10; (2) GBM: ntrees=3750, interaction.depth=2, n.minobsinnode=20; (3) survival-SVM: γ in 0.25, 0.5, 1, 2, 4; (4) Enet: α in 0.1, 0.2, 0.3, 0.4; and (5) stepwise Cox regression: direction in both, backward, forward. Five separate ML models with a total of 13 models (different hyperparameter models of the same algorithm are considered as different models) were first constructed: RSF (1 model), GBM (1 model), survival-SVM (4 hyperparameters and 4 models), Enet (4 hyperparameters and 4 models), and stepwise Cox regression (3 directions and 3 models). Second, two-by-two combinations of the above 13 models in sequential order totaled 71 models, for a total of 84 models being constructed.

Continuous features were not binarized or split-boxed during the modeling process in order to maximize the amount of information retained in the data, and for categorical features, such as sex, smoking status, and tumor staging, the modeling process used discrete values.

Model discrimination, accuracy, and clinical benefit were evaluated using the concordance index (C-index), decision curve analysis, calibration curves, and the area under the curve (AUC). Internal validation of the models was performed using the bootstrap method. Feature attribution analysis was conducted using Shapley Additive Explanations and restricted cubic splines (RCSs).

### Ethical Considerations

The protocol for this study was approved by the ethics review board of Guangzhou Medical University. All patients were informed of the data collection methods and privacy protection measures. All patient identifiers were removed after data collection and replaced with unique nonidentifiable serial numbers, and deidentified data were stored in a password-protected electronic database, with access restricted to the core research team. The deidentified data were only used for this study without sharing with third parties without additional approval.

## Results

### Baseline Characteristics of the Patients

This study enrolled 1278 eligible patients, with a median age of 61 (IQR 54-68) years, comprising 946 (74%) male and 332 (26%) female patients. Among 1278 enrolled patients with lung cancer undergoing chemotherapy, 408, 94, and 19 cases were alive at the 1-, 3-, and 5-year follow-ups, yielding overall survival rates of 31.9% (95% CI 29.4%-34.6%), 7.4% (95% CI 6%-9%), and 1.5% (95% CI 0.9%-2.4%), respectively. The specific distribution of histological types in our study population is as follows: adenocarcinoma (876/1274, 68.8%), squamous cell carcinoma (247/1274, 19.4%), small cell lung cancer (SCLC; 125/1274, 9.8%), and other types (26/1274, 2%). Some of the more important characteristics can be found in Table 1, and the complete Table 1 can be found in Multimedia Appendix 1.

**Table 1:** Baseline characteristics of chemotherapy-treated patients with lung cancer.

Variable		Missing, n (%)	Baseline data (N=1278)			Statistics		*P* value
			Living (n=758)	Deceased (n=520)				
**Sex, n (%)**		0 (0)			*χ*^2^_1_=0.6		.43	
	Male		555 (73.2)	391 (75.2)				
	Female		203 (26.8)	129 (24.8)				
Age (years), median (IQR)		0 (0)	60.0 (53.0-66.0)	63.0 (57.0-70.0)	*z*=5.5		<.01^a^	
BMI (kg/m^2^), median (IQR)		48 (3.8)	20.8 (18.9-23.0)	20.6 (18.4-22.6)	*z*=–2.1		.03^a^	
**Education, n (%)**		80 (6.3)			7.1 (2)		.03	
	Never attended school or primary school		377 (53.4)	294 (59.8)				
	Junior high school or high school		304 (43.1)	175 (35.6)				
	University and above		25 (3.5)	23 (4.7)				
WBC^b^ (10^9^ per liter), median (IQR)		150 (11.7)	8 (6-10)	8 (7-11)	*z*=2.9		<.01^a^	
HGB^c^ (g/L), median (IQR)		150 (11.7)	125.0 (111.0-136.0)	121.0 (107.0-134.0)	*z*=–3.4		<.01^a^	
D-dimer (mg/L), median (IQR)		150 (11.7)	0.69 (0.34-1.87)	0.91 (0.44-2.52)	*z*=3.7		<.01^a^	
CEA^d^ (ng/mL), median (IQR)		150 (11.7)	5 (2-20)	7 (3-52)	*z*=4.6		<.01^a^	
CA12-5^e^ (U/mL), median (IQR)		150 (11.7)	31.2 (14.7-100.8)	52.9 (19.3-145.7)	*z*=4.1		<.01^a^	
CA19-9^f^ (U/mL), median (IQR)		150 (11.7)	13.0 (7.0-26.3)	16.3 (7.2-44.9)	*z*=2.8		<.01^a^	
CYFRA21-1^g^ (ng/mL), median (IQR)		150 (11.7)	5 (3-11)	8 (4-18)	*z*=7.1		<.01^a^	
**Histology, n (%)**		4 (0.3)			*χ*^2^_3_=1.0		.79	
	LUAD^h^		522 (69.1)	354 (68.2)				
	LUSC^i^		149 (19.7)	98 (18.9)				
	SCLC^j^		69 (9.1)	56 (10.8)				
	Others		15 (2.1)	11 (2.1)				
**Tumor stage, n (%)**		31 (2.4)			*χ*^2^_1_=1.5		.22	
	T1+T2		281 (38)	176 (34.7)				
	T3+T4		458 (62)	332 (65.4)				
**Nodal stage, n (%)**		68 (5.3)			*χ*^2^_3_=16.7		<.01	
	N1		85 (11.9)	30 (6.1)				
	N2		69 (9.6)	34 (6.9)				
	N3		296 (41.3)	212 (42.9)				
	N4		266 (37.2)	218 (44.1)				
**Metastasis stage, n (%)**		156 (12.2)			*χ*^2^_1_=28.3		<.01	
	M1		174 (26.1)	59 (13)				
	M2		493 (73.9)	396 (87)				

^a^Using the Mann-Whitney *U* test.

^b^WBC: white blood cell.

^c^HGB: hemoglobin.

^d^CEA: carcinoembryonic antigen.

^e^CA12-5: carbohydrate antigen 12-5.

^f^CA19-9: carbohydrate antigen 19-9.

^g^CYFRA21-1: recombinant cytokeratin fragment antigen 21-1.

^h^LUAD: lung adenocarcinoma.

^i^LUSC: lung squamous cell carcinoma.

^j^SCLC: small cell lung cancer.

### Patient Report Outcomes Trajectory Modeling

GBTM was applied to characterize PRO trajectories. Table 2 presents detailed information on the GBTM evaluation indicators and trajectory groups. A comprehensive evaluation process is described in Tables S1-S6 in Multimedia Appendix 2. In particular, following a rigorous evaluation of model fit indices and clinical interpretability, 3 distinct trajectory groups were identified for the MDASI-LC total score (group A: high burden, group B: medium burden, and group C: low burden), 3 for the KPS score (group A: high burden, group B: medium burden, and group C: low burden), and 2 for the ZPS score (group A: high burden and group B: low burden). All equation coefficients showed significant differences (*P*<.001; Figure S1 in Multimedia Appendix 2).

**Table 2:** The number of each scale trajectory groups, classification counts, and results of various evaluation indicators for the scale^a^.

Scales	Group number	OCC^b^	Avepp^c^ (%)	*P*_j_^d^ (%)	π_j_^e^ (%)	*E*_k_^f^	BIC^g^
MDASI-LC^h^	3 (3 0 3)	33.2/33.4/81.1	98.24/90.83/93.18	62.82/22.89/14.29	62.71/22.87/14.42	0.91	–9140.61
KPS^i^	3 (0 0 0)	943.70/43.10/60.80	98.03/95.25/99.05	4.76/32.60/62.67	5.01/31.74/63.25	0.95	–6565.35
ZPS^j^	2 (0 2)	5.60/538.10	93.74/98.94	90.84/9.16	85.26/14.74	0.77	–376.79

^a^Optimization model criteria: The odds of correct classification should surpass a value of 5, reflecting a high likelihood of accurate classification. Each group should exhibit an Avepp exceeding the threshold of 0.7, indicating a high degree of confidence in the classification. The *P*_j_ should be greater than 5%, signifying that the probability of an entity belonging to a particular group is statistically significant. There should be a close alignment between *P*_j_ and the π_j_, ensuring that the posterior probabilities are congruent with the a priori probabilities. The *E*_k_ should be greater than 0.7, indicating a substantial degree of differentiation between the groups in terms of information content.

^b^OCC: odds of correct classification.

^c^Avepp: average posterior probability.

^d^*P*_j_: posterior probability of group membership.

^e^π_j_: probability of group membership.

^f^*E*_k_: relative entropy.

^g^BIC: Bayesian information criterion.

^h^MDASI-LC: MD Anderson Symptom Inventory for Lung Cancer.

^i^KPS: Karnofsky Performance Status.

^j^ZPS: Zubrod Performance Score.

### Univariate Cox Regression Analysis of Feature Selection

The missForest algorithm did not induce statistically significant changes in the data distribution after baseline imputation. A detailed comparison of the baseline data distribution before and after imputation is reported in Table S7 in Multimedia Appendix 2.

Univariate Cox regression analysis (*P* value of <.05 is statistically significant) identified 27 features significantly associated with prognosis in patients with lung cancer receiving chemotherapy, which served as an initial feature selection step. A comprehensive summary of the univariate analysis is available in Table S8 in Multimedia Appendix 2. Beyond statistically significant findings, an additional 10 features with perceived prognostic relevance, such as a family history of respiratory disease, were included in feature selection despite their lack of statistical significance in the univariate analysis. These features were retained to ensure a comprehensive evaluation of potential prognostic factors and to allow the ML models to capture complex interactions between features. Finally, 37 features were retained for the ML model development.

### Models Development and Internal Validation

**Figure 2:**
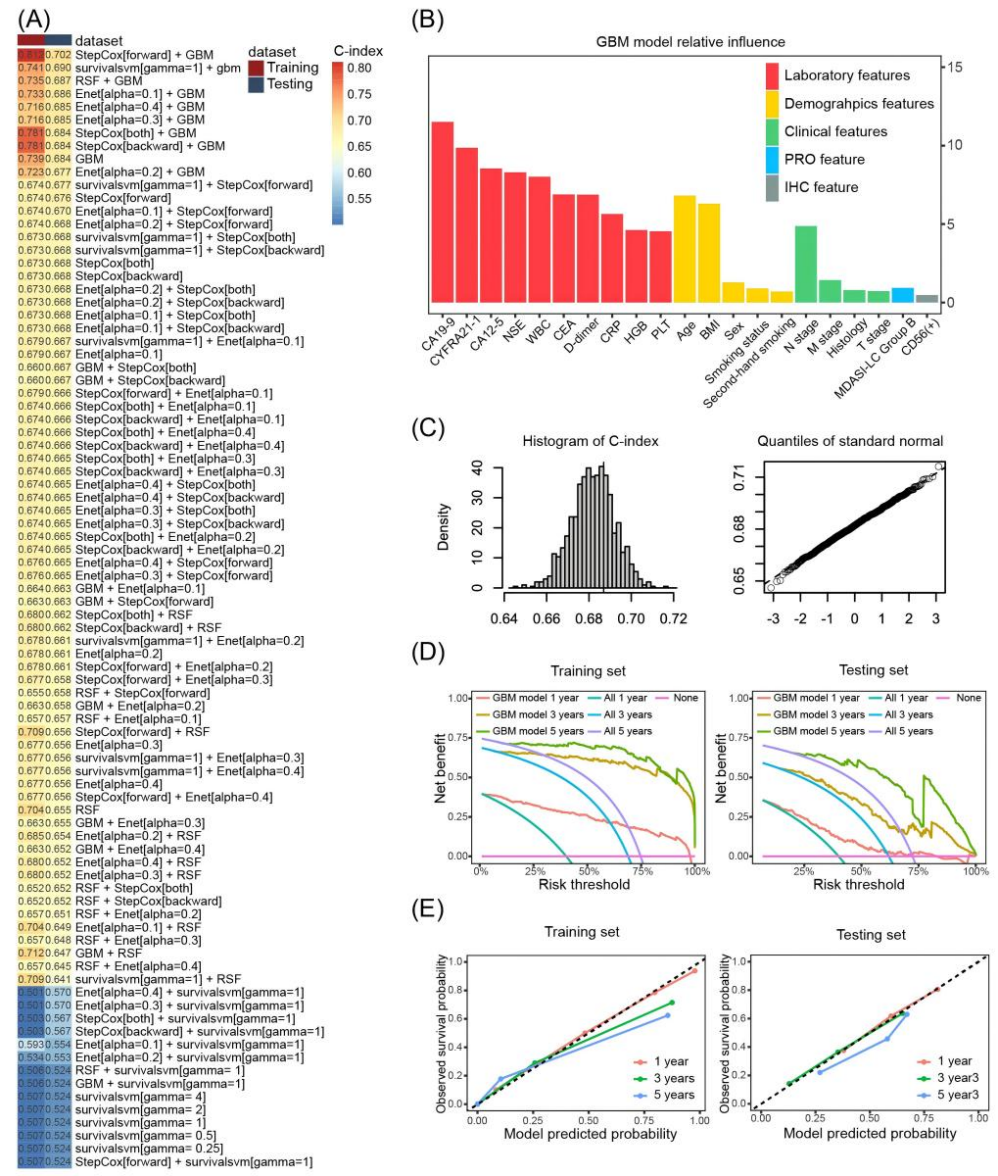
Illustration of the machine learning procedure for prediction model development and validation. (A) displays a ranking of 84 predictive models from highest to lowest based on their C-index calculated on the testing set. These models resulted from all possible 2-way combinations of 5 machine learning models. (B) presents the ranked feature importance identified by the GBM model, with the y-axis indicating the relative importance. (C) displays the C-index distribution along with results of a Shapiro-Wilk normality test, obtained during an internal validation using bootstrap resampling. (D) and (E) display DCA curves and calibration plots, respectively, for predicting 1-, 3-, and 5-year mortality risk using the optimal model on both the training and the testing set. CA12-5: carbohydrate antigen 12-5; CA19-9: carbohydrate antigen 19-9; CD56: cluster of differentiation 56; CEA: carcinoembryonic antigen; C-index: concordance index; CRP: C-reactive protein; CYFRA21-1: recombinant cytokeratin fragment antigen 21-1; DCA: decision curve analysis; Enet: elastic net; GBM: gradient boosting machine; HGB: hemoglobin; IHC: immunohistochemistry; MDASI-LC: MD Anderson Symptom Inventory for Lung Cancer; NSE: neuron-specific enolase; PLT: platelet count; PRO: patient-reported outcome; RSF: random survival forest; WBC: white blood cell.

The dataset of 1278 observations was randomly split into a training dataset (n=895) and a testing dataset (n=383) at a 7:3 ratio. Table S9 in Multimedia Appendix 2 shows that there is no difference between the distributions of the main baseline features of the patients in the training and testing sets. On the training set, a total of 84 predicted models were built with 5 ML algorithms individually or binately 5 ML models were trained and then tested both individually and in all possible combinations of pairs, yielding a total of 84 predictive models (Figure 2A).

On the testing set, we calculated the C-index of the 84 predicted models to identify the optimal predictive model. We found that the optimal predictive model was a combination of forward stepwise Cox regression and GBM and demonstrated the highest C-index of 0.702 (95% CI 0.652-0.753; Figure 2A). The final feature set of the optimal predictive model comprised 5 demographic features, 10 prechemotherapy laboratory features, 1 PRO feature, 4 clinical features, and 1 immunohistochemical feature (Figure 2B).

In total, 1000 bootstrap resamplings were performed and yielded an average C-index of 0.682 (95% CI 0.676-0.689) on the testing dataset. Shapiro-Wilk test indicated that the distribution of the C-index was normal (*P*=.86; Figure 2C). The evidence suggested a reasonable robustness of the optimal predictive model.

### Performance Evaluation of the Model

Performance evaluation of the model was implemented on both the training and testing sets. The decision curve analysis demonstrated that the predicted model had a net benefit over the strategies of predicting all deaths or all survivals when the risk thresholds were 0.40-0.97, 0.68-0.99, and 0.75-0.99 for 1-, 3-, and 5-year mortality predictions, respectively, on the training set, while 0.40-0.69, 0.62-0.99, and 0.72-0.99 on the testing set (Figure 2D).

In addition, the calibration curves displayed strong agreement between model predictions and observed outcomes (Figure 2E). The Brier scores for 1-, 3-, and 5-year mortality predictions were 0.11, 0.07, and 0.08, respectively, on the training set, while 0.20, 0.18, and 0.11 on the testing set.

**Figure 3:**
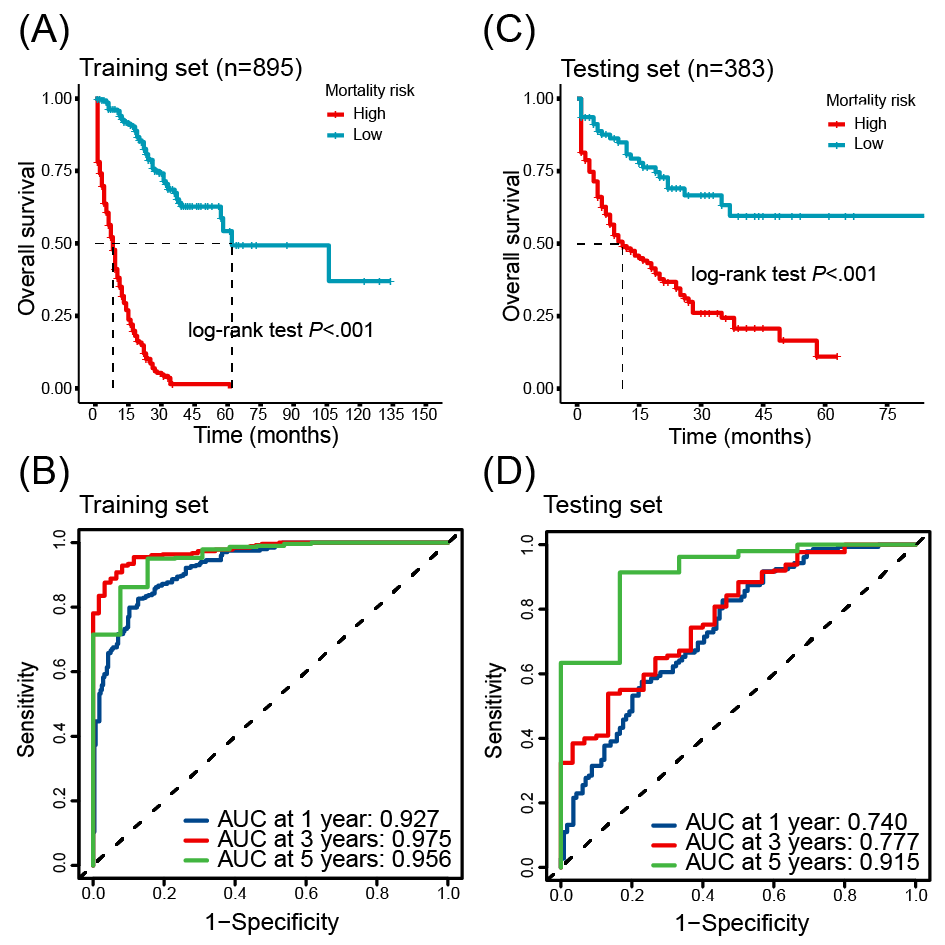
Evaluation of the mortality risk prediction model’s prognostic performance. (A) and (C) show the Kaplan-Meier curve for high- and low-risk patients, generated from the training and testing set, respectively, illustrating significant differences in survival. (B) and (D) present the time-dependent receiver operating characteristic curves assessing 1-, 3-, and 5-year mortality prediction in the training and testing data, respectively. AUC: area under the curve.

Furthermore, we determined an optimal risk score cutoff point by the *survminer* package to stratify patients into high-risk and low-risk groups. Kaplan-Meier survival analysis showed a significant difference in survival curves between the 2 groups, indicating that the model could well stratify patients when predicting 1-, 3-, or 5-year survival (*P*<.01; Figures 3A and C). The time-dependent receiver operating characteristic curve analysis found that the model demonstrated AUC values of 0.927, 0.975, and 0.956 for 1-, 3-, and 5-year mortality predictions, respectively, on the training set, while 0.740, 0.777, and 0.915 on the testing set, which exhibited robust predictive accuracy for both short-term and long-term mortality risk (Figures 3B and D).

### Feature Attribution Analysis

**Figure 4:**
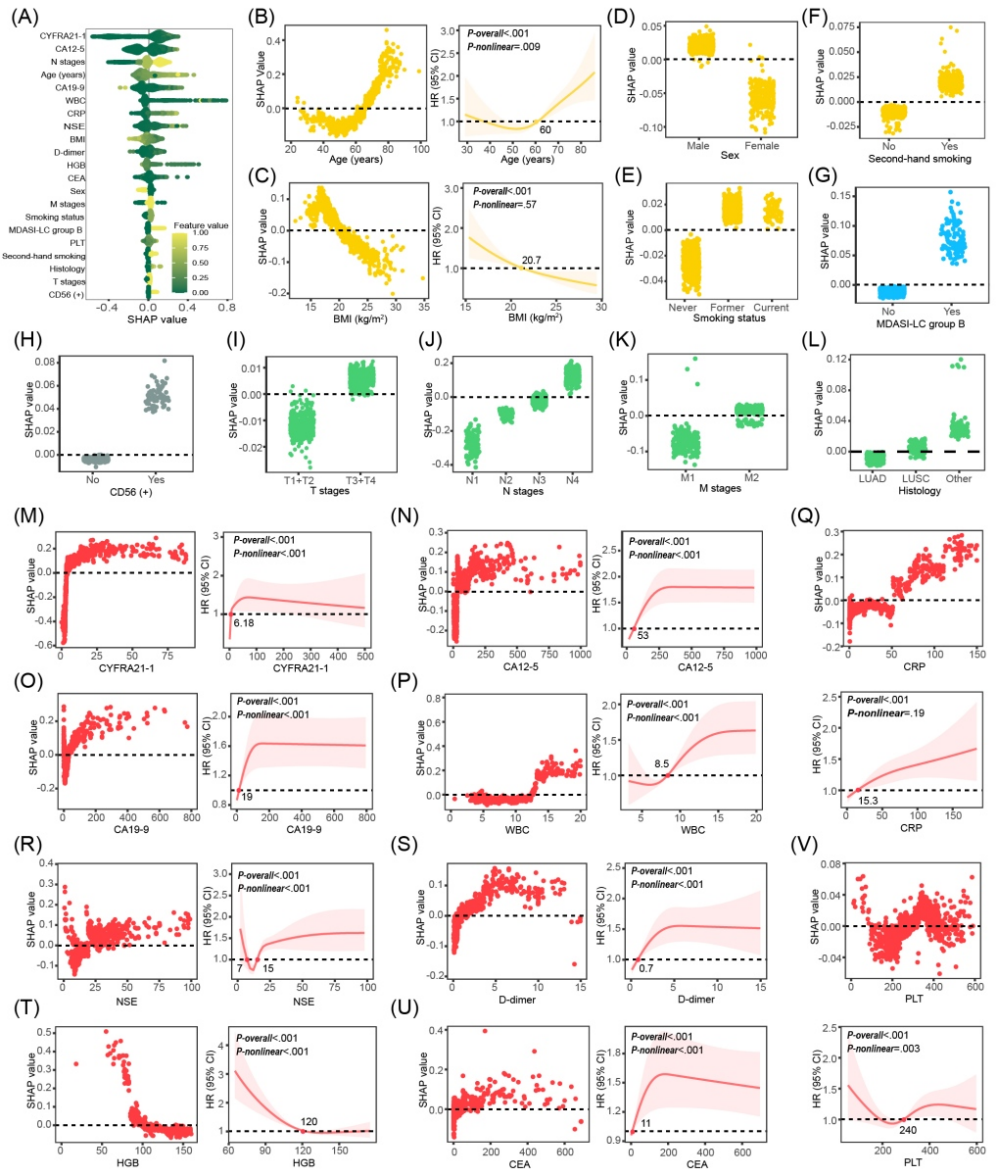
Visualization of feature importance and variable relationships influencing mortality risk. (A) displays the global feature importance. (B-F) illustrate partial dependence plots for demographic characteristics and RCS analyses for continuous variables. (G and H) and (I-L) show partial dependence plots for symptom burden PRO trajectories, immunohistochemical indicators, and tumor biological indicators, respectively. (M-V) provide partial dependence plots and RCS analysis results for prechemotherapy blood biomarkers. All partial dependence plots are annotated with the zero-SHAP value position, while the RCS plots show the points where changes in mortality risk occur. CA12-5: carbohydrate antigen 12-5; CA19-9: carbohydrate antigen 19-9; CD56: cluster of differentiation 56; CEA: carcinoembryonic antigen; CRP: C-reactive protein; CYFRA21-1: recombinant cytokeratin fragment antigen 21-1; HGB: hemoglobin; HR: hazard ratio; LUAD: lung adenocarcinoma; LUSC: lung squamous cell carcinoma; MDASI-LC: MD Anderson Symptom Inventory for Lung Cancer; NSE: neuron-specific enolase; PLT: platelet count; RCS: restricted cubic spline; SHAP: Shapley Additive Explanations; WBC: white blood cell.

Figure 4A provides insights into the most significant features influencing the model’s prediction of mortality risk in this study. These insights facilitate an improved understanding of the biological basis of risk and the identification of potential targets to optimize clinical care. The visualization of these influential features highlights their importance in guiding clinical decisions and the development of personalized treatment approaches grounded in individual patient risk profiles.

The use of RCS in this study facilitated the detection of key inflection points within continuous variables, thereby providing valuable insights into the intricate relationships among factors that contribute to mortality risk in patients with lung cancer. This refined understanding can support clinical decision-making and provide personalized approaches to patient management.

Age emerged as a significant predictor of mortality risk, with a key threshold at 60 years. Individuals younger than 60 years of age presented with a lower and less prominent mortality risk, while the risk accelerated beyond this age (Figure 4B). BMI demonstrated an inverse relationship with mortality, with a critical value of 20.7 kg/m^2^, below which mortality risk was diminished (Figure 4C). Furthermore, male sex, smoking history (inclusive of both former and current smoking), and passive smoke exposure were all linked to an increased mortality risk (Figures 4D and F).

Patients in the MDASI-LC relatively high-symptom burden group exhibited a higher mortality risk, with a median symptom score of 45.4 (IQR 36.2-51.4), while the other group had a score of 8.2 (IQR 5.2-19.8; Figure 4G). A higher chemotherapy symptom burden was associated with reduced survival.

Key clinical features, including the presence of cluster of differentiation 56 (CD56) positivity, T3 or T4 stage, N2, N3, and N4 stages, and M2 stage, were associated with a significantly higher relative risk of mortality. No significant difference in mortality risk was observed between patients with lung squamous cell carcinoma and lung adenocarcinoma, while patients with SCLC or other less frequently occurring lung cancers demonstrated decreased survival times (Figures 4H-L).

Prechemotherapy levels of laboratory markers recombinant cytokeratin fragment antigen 21-1, carbohydrate antigen 12-5, carbohydrate antigen 19-9, white blood cell count, D-dimer, and carcinoembryonic antigen showed a nonlinear increase in mortality risk, with respective critical values identified at 6.18 ng/mL, 53 U/mL, 19 U/mL, 8.5×10^9^/L, 0.7 mg/L, and 11 ng/mL, respectively. Mortality risk was significantly higher beyond these thresholds. Hemoglobin exhibited a distinct pattern, with mortality risk increasing significantly at levels below 120 g/L. In contrast, C-reactive protein was associated with a linear increase in mortality risk, with a risk inflection point at 15.3 mg/L. Prechemotherapy neuron-specific enolase levels exhibited a nonlinear relationship with mortality, where risk increased both below 7 ng/mL and above 15 ng/mL. Platelet counts also had a nonlinear relationship with mortality risk, displaying an initial decrease followed by an increase, with a turning point at 240×10^9^ per liter (Figures 4M-V).

The observed relationships are visually depicted in Shapley Additive Explanations partial dependence plots and through RCS analyses (Figures 4B-V). Subgroup analyses further confirmed these observations, demonstrating statistically significant differences in mortality risk associated with the described characteristics (*P*<.05), except for prechemotherapy platelet levels (Figure S2 in Multimedia Appendix 2 illustrating subgroup analysis validation performed after determining key values via feature attribution analysis).

## Discussion

### Principal Findings

This study developed a combined ML prediction model by integrating multisource data to assess the all-cause mortality risk of patients with lung cancer receiving chemotherapy. It identified key prognosis-related factors, including demographic characteristics, prechemotherapy laboratory indicators, longitudinal symptom burden, and clinical features, and analyzed the correlation patterns between each factor and mortality risk. The model exhibited favorable performance in discriminative ability, predictive accuracy, and clinical utility, with overall satisfactory effectiveness.

### Demographic and Lifestyle Impacts on Lung Cancer Prognosis

Age is a core factor influencing the chemotherapy outcomes of patients with lung cancer. Aging is accompanied by decreased genomic and transcriptomic stability, leading to the accumulation of mutations and abnormal gene expression, which promotes the development of malignant tumor phenotypes and reduces treatment sensitivity. Meanwhile, aging-induced metabolic disorders (such as abnormal glucose metabolism and insulin resistance) provide a favorable environment for tumor growth; the decline in immune function weakens the body’s antitumor capacity; and the reduction in tissue repair ability increases the risk of chemotherapy side effects, ultimately affecting survival [26-28]. The impact of sex on prognosis may be related to the regulation of tumor progression and chemotherapy response by sex hormones (especially estrogen), but the specific mechanism requires further research [29-31]. BMI showed a negative correlation with mortality risk, with a critical value of 20.7 kg/m^2^, and the risk increased when BMI was below this value. This finding is consistent with the “obesity paradox”—individuals with mild overweight may gain survival advantages due to the secretion of cardioprotective adipokines by adipose tissue [32-34]. In addition, smoking history (including former and current smoking) and second-hand smoke exposure significantly increased mortality risk, emphasizing the importance of smoking cessation and reducing second-hand smoke exposure for improving treatment efficacy and survival [34]. These results suggest that chemotherapy regimens should be formulated based on individual patient characteristics; nutritional status should be closely monitored during treatment with targeted support provided; and research on sex-specific treatment differences needs to be strengthened.

### The Prognostic Significance of Tumor Biological Characteristics

SCLC is often diagnosed at an advanced stage and is typically more aggressive. Tumors at T3/T4 stages (locally advanced or invasive tumors) are closely associated with increased tumor burden and local invasion, which may lead to chemotherapy resistance and elevated all-cause mortality risk [35]. The N stage reflects the spread of cancer cells to lymph nodes, leading to increased drug resistance. Patients with advanced metastatic stages (M2) frequently lose the opportunity for curative surgery, making platinum-based chemotherapy the main palliative treatment modality. However, metastatic disease, especially distant organ metastasis, is associated with inherent chemotherapy resistance, resulting in an unfavorable prognosis [36].

### Potential Prognostic Value of CD56

The association between CD56 positivity and lung cancer prognosis remains controversial. Some studies have found a correlation with poor prognosis [37,38], while others have not observed a significant association [39]. This discrepancy may be related to sample size and patient population heterogeneity, and larger-scale multicenter studies are needed to clarify the relationship between the two.

### Prognostic Value of PRO

This study suggested that the symptom burden reflected by the MDASI-LC score is related to survival time, with patients in the high-symptom burden group having a significantly increased mortality risk. This is consistent with previous research conclusions on the prognostic value of health-related quality of life [40]; incorporating MDASI-LC into routine follow-up can timely capture symptom changes and provide a basis for individualized prognostic management.

### Clinical Application Value of Biomarkers

Blood biomarkers directly reflect tumor burden and biological characteristics. Elevated prechemotherapy biomarker levels have been associated with unfavorable chemotherapy outcomes in patients with lung cancer [41-44]. For patients with abnormal biomarkers, early interventions, such as anti-inflammatory and anti-infective treatments, can improve prognosis.

### Limitations

This study has several limitations, which need to be taken into account when interpreting the results. The study used retrospective single-center data, which might limit the external validity and universality of the constructed model. Although the sample size of 1278 patients is relatively large for a single-center study, the complexity of the ML framework increases the risk of overfitting. We did not adjust the performance estimates using optimistic correction or penalty methods, which might overestimate the model’s predictive ability. The robustness of the feature attribution results was not verified under different model parameter settings, so the stability of these key prognostic thresholds still needs to be further confirmed.

Among the 1278 enrolled patients with lung cancer undergoing chemotherapy, the survival rates at 1-, 3-, and 5-year follow-ups were 31.9% (95% CI 29.4%-34.6%), 7.4% (95% CI 6.0%-9.0%), and 1.5% (95% CI 0.9%-2.4%), respectively. A smaller sample size may increase the variability of the AUC statistic. This might be part of the reason why the 5-year AUC (0.915) is higher than the 1-year (0.740) and 3-year (0.777) AUC, which needs to be taken into account when interpreting the long-term predictive performance of the model.

In this study, 84 models were constructed on the training set, and then, the C-index was used on the test set to determine the best model. This process may violate the principle that the test set should only be used once to evaluate the performance of the final, prespecified model, which may lead to an overestimation of the model’s C-index. In future research, model selection and hyperparameter tuning can be attempted to be accomplished solely through cross-validation of the training set, with the test set reserved only for evaluating the prespecified final model performance.

### Conclusions

This study developed an innovative ML model to predict all-cause mortality risk in patients with lung cancer receiving chemotherapy. The model demonstrates acceptable discrimination, accuracy, and clinical efficacy, offering clinicians an interpretable tool for mortality risk assessment. By enabling personalized risk stratification, this model can guide targeted postchemotherapy care for patients with high risk, ultimately supporting the development of individualized treatment plans.

## Data Availability

The datasets generated or analyzed during this study are not publicly available due patient privacy but are available from the corresponding author on reasonable request.

## Appendix

Multimedia Appendix 1 Complete baseline characteristics of chemotherapy-treated patients with lung cancer.

Multimedia Appendix 2 The evaluation of trajectory model fitting for the MD Anderson Symptom Inventory for Lung Cancer, Karnofsky Performance Status, and Zubrod Performance Score scales, the comparison of baseline data distribution before and after missForest interpolation, the results of univariate Cox regression, the comparison of baseline characteristics between the training and test sets, and the subgroup analysis of key values for feature attribution.

## Abbreviations

AUC: area under the curve
CD56: cluster of differentiation 56
C-index: concordance index
Enet: elastic net
GBM: gradient boosting machine
GBTM: group-based trajectory modeling
KPS: Karnofsky Performance Status
MDASI-LC: MD Anderson Symptom Inventory for Lung Cancer
ML: machine learning
PRO: patient-reported outcome
RCS: restricted cubic spline
RSF: random survival forest
SCLC: small cell lung cancer
Survival-SVM: survival support vector machine
ZPS: Zubrod Performance Score
