# Supplementary material for: A Machine Learning Approach to Predicting Mortality Risk in Chemotherapy-Treated Lung Cancer: Machine Learning Model Development and Validation
Source: JMIR Med Inform. 2025 Dec 18;13:e72424. doi: 10.2196/72424 (PMC12757710; doi:10.2196/72424)
Supplement: Multimedia Appendix 1 [file medinform_v13i1e72424_app1.docx]

| Variable | Missing  n (%) | Baseline data(N = 1,278) | | Statistic | *P* value |
| --- | --- | --- | --- | --- | --- |
|  |  | Living  (n = 758)  n(%) | Deceased  (n = 520)  n(%) |  |  |
| **Sex** | 0(0) |  |  |  |  |
| Male |  | 555(73.2) | 391(75.2) | *χ^2^*=0.6(1) | .43 |
| Female |  | 203(26.8) | 129(24.8) |  |  |
| **Age[*M*(*P*_25_,*P*_75_)]** | 0(0) | 60.0(53.0,66.0) | 63.0(57.0,70.0) | *Z*=5.5 | **<.01^b^** |
| **BMI[*M*(*P*_25_,*P*_75_)]** | 48(3.8) | 20.8(18.9,23.0) | 20.6(18.4,22.6) | *Z*=-2.1 | **.03^b^** |
| **Education** | 80(6.3) |  |  |  |  |
| Never attended school/Primary school |  | 377(53.4) | 294(59.8) | *χ^2^*=7.1(2) | **.03** |
| Junior High School/High School |  | 304(43.1) | 175(35.6) |  |  |
| University and above |  | 25(3.5) | 23(4.7) |  |  |
| **Respiratory Disease History** | 0(0) |  |  |  |  |
| No |  | 651(85.9) | 437(84.0) | *χ^2^*=0.9(1) | .36 |
| Yes |  | 107(14.1) | 83(16.0) |  |  |
| **Chronic Gastritis** | 13(1.0) |  |  |  |  |
| No |  | 738(98.8) | 508(98.1) | *χ^2^*=1.1(1) | .30 |
| Yes |  | 9(1.2) | 10(1.9) |  |  |
| **Chronic Hepatitis** | 13(1.0) |  |  |  |  |
| No |  | 726(97.2) | 500(96.5) | *χ^2^*=0.5(1) | .50 |
| Yes |  | 21(2.8) | 18(3.5) |  |  |
| **Family History of Cancer** | 56(4.4) |  |  |  |  |
| No |  | 701(97.0) | 475(95.2) | *χ^2^*=2.5(1) | .11 |
| Yes |  | 22(3) | 24(4.8) |  |  |
| **Smoking Status** | 0(0) |  |  |  |  |
| Never smoker |  | 301(39.7) | 201(38.7) | *χ^2^*=2.0(2) | .38 |
| Former smoker |  | 408(53.8) | 294(56.5) |  |  |
| Current smoker |  | 49(6.0) | 25(4.8) |  |  |
| **SHS** | 0(0) |  |  |  |  |
| No |  | 525(69.3) | 340(65.4) | *χ^2^*=2.1(1) | .15 |
| Yes |  | 233(30.7) | 180(34.6) |  |  |
| **SHS from Parents** | 0(0) |  |  |  |  |
| No |  | 163(70.0) | 131(72.8) | *χ^2^*=0.4(1) | .53 |
| Yes |  | 70(30.0) | 49(27.2) |  |  |
| **SHS from Spouse** | 0(0) |  |  |  |  |
| No |  | 163(70.0) | 125(69.4) | *χ^2^*=0.01(1) | .91 |
| Yes |  | 70(30.0) | 55(30.6) |  |  |
| **SHS from Offspring** | 0(0) |  |  |  |  |
| No |  | 122(52.4) | 97(53.8) | *χ^2^*=0.1(1) | .76 |
| Yes |  | 111(47.6) | 83(46.1) |  |  |
| **SHS from Friends or Colleagues** | 0(0) |  |  |  |  |
| No |  | 174(74.7) | 136(75.6) | *χ^2^*=0.04(1) | .84 |
| Yes |  | 59(25.3) | 44(24.4) |  |  |
| **Housing Ventilation** | 19(1.5) |  |  |  |  |
| Good |  | 672(90.3) | 466(90.5) | *χ^2^*=0.01(1) | .92 |
| Poor |  | 72(9.7) | 49(9.5) |  |  |
| **Availability of an Exhaust Fan** | 25(2.0) |  |  |  |  |
| No |  | 25(3.4) | 25(4.9) | *χ^2^*=1.7(1) | .19 |
| Yes |  | 714(96.6) | 489(95.1) |  |  |
| **Biomass Fuel Exposure** | 29(2.3) |  |  |  |  |
| No |  | 723(97.7) | 489(96.1) | *χ^2^*=2.8(1) | .10 |
| Yes |  | 17(2.3) | 20(3.9) |  |  |
| **Whether to prepare meals** | 16(1.3) |  |  |  |  |
| No |  | 12(1.6) | 5(1.0) | *χ^2^*=3.9(2) | .14 |
| Occasionally |  | 345(46.4) | 267(51.5) |  |  |
| Frequently |  | 387(52.0) | 246(47.5) |  |  |
| **Frequency of fruit and vegetable intake** | 16(1.3) |  |  |  |  |
| No consumption/1-2 days per week |  | 61(8.2) | 49(9.5) | *χ^2^*=1.9(2) | .39 |
| 3-4 days per week |  | 457(61.3) | 326(63.2) |  |  |
| 5-7 days per week |  | 228(30.6) | 141(27.3) |  |  |
| **Frequency of smoked meat intake** | 17(1.3) |  |  |  |  |
| No consumption/1-2 days per week |  | 20(2.7) | 12(2.3) | *χ^2^*=2.3(2) | .32 |
| 3-4 days per week |  | 632(84.8) | 453(87.8) |  |  |
| 5-7 days per week |  | 93(12.5) | 51(9.9) |  |  |
| **Frequency of salted vegetables intake** | 18(1.4) |  |  |  |  |
| No consumption/1-2 days per week |  | 167(22.5) | 124(24.0) | *χ^2^*=0.5(2) | .80 |
| 3-4 days per week |  | 520(69.9) | 352(68.2) |  |  |
| 5-7 days per week |  | 57(7.7) | 40(7.8) |  |  |
| **Frequency of salted fish and cured meats intake** | 19(1.5) |  |  |  |  |
| No consumption/1-2 days per week |  | 145(19.5) | 103(20.0) | *χ^2^*=0.4(2) | .82 |
| 3-4 days per week |  | 558(74.9) | 386(75.1) |  |  |
| 5-7 days per week |  | 42(5.6) | 25(4.9) |  |  |
| **WBC(10^9^/L)[*M*(*P*_25_,*P*_75_)]** | 150(11.7) | 8(6,10) | 8(7,11) | *Z*=2.9 | **<.01^b^** |
| **PLT(10^9^/L)[*M*(*P*_25_,*P*_75_)]** | 150(11.7) | 286.0(225.0,358.0) | 297.5(223.0,365.0) | *Z*=1.0 | .32^b^ |
| **HGB(g/L)[*M*(*P*_25_,*P*_75_)]** | 150(11.7) | 125.0(111.0,136.0) | 121.0(107.0,134.0) | *Z*=-3.4 | **<.01^b^** |
| **D-dimer(mg/L)[*M*(*P*_25_,*P*_75_)]** | 150(11.7) | 0.69(0.34,1.87) | 0.91(0.44,2.52) | *Z*=3.7 | **<.01^b^** |
| **CD4+[*M*(*P*_25_,*P*_75_)]** | 150(11.7) | 662.0(460.0,856.0) | 596.0(424.0,824.0) | *Z*=-1.6 | .06^b^ |
| **CD8+[*M*(*P*_25_,*P*_75_)]** | 150(11.7) | 440.0(300.0,592.0) | 406.0(300.0,576.0) | *Z*=-0.9 | .38^b^ |
| **CD3+[*M*(*P*_25_,*P*_75_)]** | 150(11.7) | 1192.0(828.0,1540.0) | 1076.0(820.0,1440.0) | *Z*=-1.7 | .09**^b^** |
| **CD4+/CD8+[*M*(*P*_25_,*P*_75_)]** | 150(11.7) | 1.49(1.11,1.98) | 1.46(1.03,1.92) | *Z*=-1.3 | .21**^b^** |
| **CRP(mg/L)[*M*(*P*_25_,*P*_75_)]** | 150(11.7) | 12.2(2.5,40.3) | 18.9(4.4,58.2) | *Z*=2.7 | **.01^b^** |
| **GLU(mmol/L)[*M*(*P*_25_,*P*_75_)]** | 150(11.7) | 6.31(5.30,8.17) | 6.42(5.40,8.08) | *Z*=0.5 | .60**^b^** |
| **UA(umol/L)[*M*(*P*_25_,*P*_75_)]** | 150(11.7) | 322.5(254.0,393.5) | 312.0(246.5,390.0) | *Z*=-1.1 | .29**^b^** |
| **ESR(mm/h)[*M*(*P*_25_,*P*_75_)]** | 150(11.7) | 40.5(23.0,61.0) | 51.0(24.0,64.0) | *Z*=0.6 | .53**^b^** |
| **CEA(ng/ml)[*M*(*P*_25_,*P*_75_)]** | 150(11.7) | 5(2,20) | 7(3,52) | *Z*=4.6 | **<.01^b^** |
| **CA12-5(U/ml)[*M*(*P*_25_,*P*_75_)]** | 150(11.7) | 31.2(14.7,100.8) | 52.9(19.3,145.7) | *Z*=4.1 | **<.01^b^** |
| **CA19-9(U/ml)[*M*(*P*_25_,*P*_75_)]** | 150(11.7) | 13.0(7.0,26.3) | 16.3(7.2,44.9) | *Z*=2.8 | **<.01^b^** |
| **NSE(ng/ml)[*M*(*P*_25_,*P*_75_)]** | 150(11.7) | 13.9(11.0,20.7) | 15.9(11.9,27.5) | *Z*=3.9 | **<.01^b^** |
| **CYFRA21-1(ng/ml)[*M*(*P*_25_,*P*_75_)]** | 150(11.7) | 5(3,11) | 8(4,18) | *Z*=7.1 | **<.01^b^** |
| **Location of lung cancer** | 31(2.4) |  |  |  |  |
| Both lungs |  | 4(0.5) | 1(0.2) | — | .61**^a^** |
| Right lung |  | 411(55.5) | 289(57.1) |  |  |
| Left lung |  | 326(44.0) | 216(42.7) |  |  |
| **Histology** | 4(0.3) |  |  |  |  |
| LUAD |  | 522(69.1) | 354(68.2) | *χ^2^*=1.0(3) | .79 |
| LUSC |  | 149(19.7) | 98(18.9) |  |  |
| SCLC |  | 69(9.1) | 56(10.8) |  |  |
| Others |  | 15(2.1) | 11(2.1) |  |  |
| **Tumor stage** | 31(2.4) |  |  |  |  |
| T1 + T2 |  | 281(38.0) | 176(34.7) | *χ^2^*=1.5(1) | .22 |
| T3 + T4 |  | 458(62.0) | 332(65.4) |  |  |
| **Nodal stage** | 68(5.3) |  |  |  |  |
| N1 |  | 85(11.9) | 30(6.1) | *χ^2^*=16.7(3) | **<.01** |
| N2 |  | 69(9.6) | 34(6.9) |  |  |
| N3 |  | 296(41.3) | 212(42.9) |  |  |
| N4 |  | 266(37.2) | 218(44.1) |  |  |
| **Metastasis stage** | 156(12.2) |  |  |  |  |
| M1 |  | 174(26.1) | 59(13.0) | *χ^2^*=28.3(1) | **<.01** |
| M2 |  | 493(73.9) | 396(87.0) |  |  |
| **Stage** | 3(0.2) |  |  |  |  |
| Stage I-II |  | 74(9.8) | 19(3.7) | *χ^2^*=17.2(1) | **<.01** |
| Stage III-IV |  | 681(90.2) | 501(96.4) |  |  |

^a^ using Fisher's exact test

^b^ using the Mann-Whitney U test

Abbreviations: P_25_,the 25th percentiles;P_75_,the 75th percentiles;BMI, Body Mass Index; SHS, second-hand smoking; WBC, White Blood Cell; PLT, Platelet Count; HGB, Hemoglobin; CD4+, CD4-Positive T-Lymphocytes; CD8+,CD8-Positive T-Lymphocytes; CD3+,CD3-Positive T-Lymphocytes; CD4+/CD8+, Ratio of CD4-Positive to CD8-Positive T-Lymphocytes; CRP,C-Reactive Protein; GLU, Glucose; UA, Uric Acid; ESR, Erythrocyte Sedimentation Rate; CEA, Carcinoembryonic Antigen; CA12-5,Cancer Antigen 125, CA19-9,Cancer Antigen 19-9; NSE, Neuron-Specific Enolase; CYFRA21-1,Cytokeratin-1 Fragment; LUAD, Lung Adenocarcinoma; LUSC, Lung Squamous Cell Carcinoma; SCLC, Small Cell Lung Cancer.
