# Supplementary material for: A Machine Learning Approach to Predicting Mortality Risk in Chemotherapy-Treated Lung Cancer: Machine Learning Model Development and Validation
Source: JMIR Med Inform. 2025 Dec 18;13:e72424. doi: 10.2196/72424 (PMC12757710; doi:10.2196/72424)
Supplement: Multimedia Appendix 2 [file medinform_v13i1e72424_app2.docx]

**Table S1.** Evaluation metrics for the fitting effects of different trajectory models for MDASI-LC.

| Group | *Avepp(%)* | *OCC* | *P_j_(%)* | *π_j_(%)* | *BIC^#2^* | *E_k_* |
| --- | --- | --- | --- | --- | --- | --- |
| *1Group (3 )* | 100.000 | . | 100.000 | 100.000 | -10075.00 | 0.000 |
| *2Group (3 3 )* | 99.138/98.384 | 55.8/125.5 | 67.399/32.601 | 67.345/32.655 | -9283.13 | 0.957 |
| *3Group (3 0 3 )* | 98.241/90.825/93.184 | 33.2/33.4/81.1 | 62.821/22.894/14.286 | 62.705/22.872/14.424 | -9140.61 | 0.911 |
| *4Group (3 0 3 3 )* | 94.673/86.446/92.921/89.682 | 14.6/27.8/53.9/118.5 | 56.410/17.216/19.414/6.960 | 54.922/18.666/19.576/6.836 | -9097.97 | 0.874 |
| *5Group (3 0 1 3 2 )* | 91.189/82.573/88.058/86.492/93.520 | 15.9/12.7/39.5/41.8/307.2 | 38.828/27.839/15.385/13.736/4.212 | 39.400/27.116/15.715/13.282/4.487 | -9063.75 | 0.818 |

Abbreviations: Odds of Correct Classification; Avepp, Average Posterior Probability; P_j_, Posterior Probability of Group Membership; π_j_, Probability of Group Membership; BIC, Bayesian Information Criterion; E_k_, Relative Entropy.

**Table S2.** Evaluation of the fitting performance for each trajectory group within the group trajectory mode for MDASI-LC.

| Group | Class | Parameter | *β* | *SE* | *t* | *P* |
| --- | --- | --- | --- | --- | --- | --- |
| *3Group* | Class.1 | Intercept | 21.93618 | 0.62092 | 35.329 | <.001 |
|  |  | Linear | 9.63373 | 1.55713 | 6.187 | <.001 |
|  |  | Quadratic | -5.43358 | 1.02758 | -5.288 | <.001 |
|  |  | Cubic | 0.80511 | 0.17401 | 4.627 | <.001 |
|  | Class.2 | Intercept | 50.43067 | 0.98447 | 51.226 | <.001 |
|  | Class.3 | Intercept | 65.28027 | 1.44171 | 45.280 | <.001 |
|  |  | Linear | 21.45185 | 3.62292 | 5.921 | <.001 |
|  |  | Quadratic | -11.82588 | 2.40532 | -4.917 | <.001 |
|  |  | Cubic | 1.63786 | 0.41237 | 3.972 | <.001 |
| Significant level was set at *P*≤.05. | | | | | | |

**Table S3.** Evaluation metrics for the fitting effects of different trajectory models for KPS scale.

| **Group** | ***Avepp(%)*** | ***OCC*** | ***P_j_(%)*** | ***π_j_(%)*** | ***BIC^#2^*** | ***E_k_*** |
| --- | --- | --- | --- | --- | --- | --- |
| ***1Group (1 )*** | 100.000 |  | 100.000 | 100.000 | -7191.77 | 0.000 |
| ***2Group (0 0 )*** | 92.569-99.188 | 25.4-60.0 | 34.982-65.018 | 32.910-67.090 | -6755.91 | 0.896 |
| ***3Group (0 0 0 )*** | 98.031-95.247-99.053 | 943.7-43.1-60.8 | 4.762-32.601-62.637 | 5.012-31.738-63.250 | -6565.35 | 0.946 |
| ***4Group (0 0 0 0 )*** | 98.096-95.165-98.978-99.739 | 975.6-42.4-56.7-148413.1 | 4.762-32.601-62.454-0.183 | 5.017-31.679-63.047-0.257 | -6568.17 | 0.956 |
| ***5Group (0 0 0 0 0 )*** | 91.957-85.955-94.861-98.837-99.815 | 386.3-187.3-41.3-50.3-203244.2 | 2.930-2.747-31.685-62.454-0.183 | 2.875-3.163-30.908-62.789-0.265 | -6564.30 | 0.950 |

Abbreviations: Odds of Correct Classification; Avepp, Average Posterior Probability; P_j_, Posterior Probability of Group Membership; π_j_, Probability of Group Membership; BIC, Bayesian Information Criterion; E_k_, Relative Entropy.

**Table S4.** Evaluation of the fitting performance for each trajectory group within the group trajectory mode for KPS scale.

| **Group** | **Class** | **Parameter** | ***β*** | ***SE*** | ***t*** | ***P*** |
| --- | --- | --- | --- | --- | --- | --- |
| ***3Group*** | Class.1 | Intercept | 70.70644 | 0.48984 | 144.346 | <.001 |
|  | Class.2 | Intercept | 81.56879 | 0.17532 | 465.257 | <.001 |
|  | Class.3 | Intercept | 89.60291 | 0.11450 | 782.558 | <.001 |
| Significant level was set at P≤.05. | | | | | | |

**Table S5.** Evaluation metrics for the fitting effects of different trajectory models for ZPS scale.

| **Group** | ***Avepp(%)*** | ***OCC*** | ***P_j_(%)*** | ***π_j_(%)*** | ***BIC^#2^*** | ***E_k_*** |
| --- | --- | --- | --- | --- | --- | --- |
| ***1Group (0)*** | 100.000 |  | 100.000 | 100.000 | -717.89 | 0.000 |
| ***2Group (0 2)*** | 93.744-98.937 | 5.6-538.1 | 90.842-9.158 | 85.257-14.743 | -376.79 | 0.770 |
| ***3Group (/ )*** | / | / | / | / | / | / |
| ***4Group (/ )*** | / | / | / | / | / | / |
| ***5Group (/ )*** | / | / | / | / | / | / |

"/" indicates that the data could not be fitted

Abbreviations: Odds of Correct Classification; Avepp, Average Posterior Probability; P_j_, Posterior Probability of Group Membership; π_j_, Probability of Group Membership; BIC, Bayesian Information Criterion; E_k_, Relative Entropy.

**Table S6.** Evaluation of the fitting performance for each trajectory group within the group trajectory mode for ZPS scale.

| **Group** | **Class** | **Parameter** | ***β*** | ***SE*** | ***t*** | ***P*** |
| --- | --- | --- | --- | --- | --- | --- |
| ***2Group*** | Class.1 | Intercept | 1.04566 | 0.00868 | 120.468 | <.001 |
|  | Class.2 | Intercept | 1.74576 | 0.04661 | 37.455 | <.001 |
|  |  | Linear | 0.21981 | 0.05623 | 3.909 | <.001 |
|  |  | Quadratic | -0.05108 | 0.01500 | -3.405 | <.001 |
| Significant level was set at *P*≤.05. | | | | | | |

**Table S7.**A comparative analysis of baseline data distribution before and after imputation using missForest.

| **Variable** | **Imputed baseline data** | | **Statistic** | **P-Value** |
| --- | --- | --- | --- | --- |
|  | **Before imputation**  **(n=1278)** | **After imputation**  **(n=1278)** |  |  |
| **BMI[*M*(*P*_25_,*P*_75_)]** | 20.8(18.7,22.9) | 20.8(18.7,22.9) | *Z*=-0.06 | .955 ^a^ |
| **Education** |  |  |  |  |
| Never attended school/Primary school | 671(56.01) | 743(58.14) | *χ^2^*=1.15 | .563 |
| Junior High School/High School | 479(39.98) | 487(38.11) |  |  |
| University and above | 48(4.01) | 48(3.76) |  |  |
| **Availability of an Exhaust Fan** |  |  |  |  |
| No | 50(3.99) | 50(3.91) | *χ^2^*=0.01 | .920 |
| Yes | 1203(96.01) | 1228(96.09) |  |  |
| **Frequency of fruit and vegetable intake** |  |  |  |  |
| No consumption/1-2 days per week | 110(8.72) | 110(8.61) | *χ^2^*=0.06 | .970 |
| 3-4 days per week | 783(62.04) | 799(62.52) |  |  |
| 5-7 days per week | 369(29.24) | 369(28.87) |  |  |
| **WBC(10^9^/L)[*M*(*P*_25_,*P*_75_)]** | 8(7,11) | 8(7,11) | *Z*=-0.23 | .819 ^a^ |
| **PLT(10^9^/L)[*M*(*P*_25_,*P*_75_)]** | 289.0(225.0,362.0) | 290.0(227.0,364.0) | *Z*=-0.31 | .756 ^a^ |
| **HGB(g/L)[*M*(*P*_25_,*P*_75_)]** | 123.0(110.0,135.0) | 123.0(110.0,135.0) | *Z*=-0.16 | .875 ^a^ |
| **D-dimer(mg/L)[*M*(*P*_25_,*P*_75_)]** | 0.78(0.36,2.10) | 0.77(0.37,2.13) | *Z*=-0.31 | .755 ^a^ |
| **CRP(mg/L)[*M*(*P*_25_,*P*_75_)]** | 14.6(3.3,46.0) | 14.5(2.2,44.7) | *Z*=1.14 | .253 ^a^ |
| **CEA(ng/ml)[*M*(*P*_25_,*P*_75_)]** | 6(3,28) | 6(3,31) | *Z*=-0.95 | .343 ^a^ |
| **CA12-5(U/ml)[*M*(*P*_25_,*P*_75_)]** | 40.7(16.0,117.6) | 41.4(16.4,124.7) | *Z*=-0.77 | .442 ^a^ |
| **CA19-9(U/ml)[*M*(*P*_25_,*P*_75_)]** | 14.2(7.0,31.1) | 14.0(6.5,31.4) | *Z*=0.40 | .687 ^a^ |
| **NSE(ng/ml)[*M*(*P*_25_,*P*_75_)]** | 14.8(11.3,23.5) | 15.0(11.4,24.6) | *Z*=-0.76 | .445 ^a^ |
| **CYFRA21-1(ng/ml)[*M*(*P*_25_,*P*_75_)]** | 6(3,13) | 6(3,14) | *Z*=-0.70 | .486 ^a^ |
| **Location of lung cancer** |  |  |  |  |
| Both lungs | 5(0.40) | 5(0.39) | *χ^2^*=0.21 | .900 |
| Right lung | 700(56.13) | 729(57.04) |  |  |
| Left lung | 542(43.46) | 544(42.57) |  |  |
| **Types of lung cancer** |  |  |  |  |
| Lung Adenocarcinoma | 876(68.76) | 879(68.78) | *χ^2^*=0.00 | .998 |
| Lung Squamous Cell Carcinoma | 247(19.39) | 248(19.41) |  |  |
| Small Cell Lung Cancer | 125(9.81) | 125(9.78) |  |  |
| Others | 26(2.04) | 26(2.03) |  |  |
| **Tumor stage** |  |  |  |  |
| T1 + T2 | 457(36.65) | 461(36.07) | *χ^2^*=0.09 | .764 |
| T3 + T4 | 790(63.35) | 817(63.93) |  |  |
| **Nodal stage** |  |  |  |  |
| N1 | 115(9.50) | 131(10.25) | *χ^2^*=0.53 | .912 |
| N2 | 103(8.51) | 104(8.14) |  |  |
| N3 | 508(41.98) | 540(42.25) |  |  |
| N4 | 484(40.00) | 503(39.36) |  |  |
| **Metastasis stage** |  |  |  |  |
| M1 | 233(20.77) | 255(19.95) | *χ^2^*=0.24 | .621 |
| M2 | 889(79.23) | 1023(80.05) |  |  |
| **Stage** |  |  |  |  |
| Stage I+ II | 93(7.29) | 93(7.28) | *χ^2^*=0.00 | .987 |
| Stage III+ IV | 1182(92.71) | 1185(92.72) |  |  |

^a^ using the Mann-Whitney U test.

Abbreviations: BMI, Body Mass Index; WBC, White Blood Cell; PLT, Platelet Count; HGB, Hemoglobin; CD4+, CD4-Positive T-Lymphocytes. CD8+, CD8-Positive T-Lymphocytes. CD3+, CD3-Positive T-Lymphocytes. CD4+/CD8+, Ratio of CD4-Positive to CD8-Positive T-Lymphocytes. CRP, C-Reactive Protein. GLU, Glucose. UA, Uric Acid. ESR, Erythrocyte Sedimentation Rate. CEA, Carcinoembryonic Antigen. CA12-5, Cancer Antigen 125, CA19-9, Cancer Antigen 19-9. NSE, Neuron-Specific Enolase. CYFRA21-1, Cytokeratin-1 Fragment .LUAD, Lung Adenocarcinoma. LUSC, Lung Squamous Cell Carcinoma. SCLC, Small Cell Lung Cancer.

**Table S8.** Results of univariate Cox regression analysis.

| **Characteristics** | **Coefficients** | **SE** | **Statistic** | ***P*-value** | **HR (95%CI)** |
| --- | --- | --- | --- | --- | --- |
| **Sex** |  |  |  |  |  |
| Female |  |  |  |  | 1.00(ref) |
| Male | 0.263 | 0.102 | 6.682 | **.009** | 1.30(1.07~1.59) |
| **Age** | 0.020 | 0.004 | 24.344 | **<.001** | 1.02(1.01~1.03) |
| **BMI** | -0.074 | 0.015 | 24.367 | **<.001** | 0.93(0.90~0.96) |
| **Education** |  |  |  |  |  |
| Never attended school/Primary school |  |  |  |  | 1.00(ref) |
| Junior High School/High School | -0.261 | 0.096 | 7.454 | **.006** | 0.77(0.64~0.93) |
| University and above | -0.083 | 0.217 | 0.145 | .703 | 0.92(0.60~1.41) |
| **Respiratory Disease History** |  |  |  |  |  |
| No |  |  |  |  | 1.00(ref) |
| Yes | 0.230 | 0.120 | 3.667 | .056 | 1.26(0.99~1.59) |
| **Family History of Cancer** |  |  |  |  |  |
| No |  |  |  |  | 1.00(ref) |
| Yes | 0.206 | 0.209 | 0.973 | .324 | 1.23(0.82~1.85) |
| **Smoking Status** |  |  |  |  |  |
| Never |  |  |  |  | 1.00(ref) |
| Former smoker | 0.220 | 0.092 | 5.741 | **.016** | 1.25(1.04~1.49) |
| Current smoker | 0.075 | 0.212 | 0.126 | .723 | 1.08(0.71~1.63) |
| **Second-han smoking(SSE)** |  |  |  |  |  |
| No |  |  |  |  | 1.00(ref) |
| Yes | 0.184 | 0.092 | 3.987 | **.045** | 1.20(1.00~1.44) |
| **SSE from Parents** |  |  |  |  |  |
| No |  |  |  |  | 1.00(ref) |
| Yes | 0.003 | 0.150 | 0.000 | .987 | 1.00(0.75~1.35) |
| **SSE from Spouse** |  |  |  |  |  |
| No |  |  |  |  | 1.00(ref) |
| Yes | 0.004 | 0.143 | 0.001 | .980 | 1.00(0.76~1.33) |
| **SSE from Offspring** |  |  |  |  |  |
| No |  |  |  |  | 1.00(ref) |
| Yes | 0.319 | 0.120 | 7.055 | **.007** | 1.38(1.09~1.74) |
| **SSE from Friends or Colleagues** |  |  |  |  |  |
| No |  |  |  |  | 1.00(ref) |
| Yes | 0.097 | 0.158 | 0.380 | .538 | 1.10(0.81~1.50) |
| **Housing Ventilation** |  |  |  |  |  |
| Good |  |  |  |  | 1.00(ref) |
| Poor | 0.246 | 0.151 | 2.670 | .102 | 1.28(0.95~1.72) |
| **Availability of an Exhaust Fan** |  |  |  |  |  |
| No |  |  |  |  | 1.00(ref) |
| Yes | -0.686 | 0.206 | 11.091 | **<.001** | 0.50(0.34~0.75) |
| **Biomass Fuel Exposure** |  |  |  |  |  |
| No |  |  |  |  | 1.00(ref) |
| Yes | 0.680 | 0.229 | 8.843 | **.002** | 1.97(1.26~3.09) |
| **Whether to prepare meals** |  |  |  |  |  |
| No |  |  |  |  | 1.00(ref) |
| Occasionally | -0.301 | 0.453 | 0.441 | .507 | 0.74(0.30~1.80) |
| Frequently | -0.540 | 0.453 | 1.418 | .234 | 0.58(0.24~1.42) |
| **Frequency of fruit and vegetable intake** |  |  |  |  |  |
| No consumption/1-2 days per week |  |  |  |  | 1.00(ref) |
| 3-4 days per week | -0.285 | 0.154 | 3.433 | .064 | 0.75(0.56~1.02) |
| 5-7 days per week | -0.378 | 0.166 | 5.166 | **.023** | 0.69(0.50~0.95) |
| **Frequency of smoked meat intake** |  |  |  |  |  |
| No consumption/1-2 days per week |  |  |  |  | 1.00(ref) |
| 3-4 days per week | 0.144 | 0.293 | 0.242 | .623 | 1.15(0.65~2.05) |
| 5-7 days per week | 0.054 | 0.321 | 0.028 | .867 | 1.06(0.56~1.98) |
| **Frequency of salted vegetables intake** |  |  |  |  |  |
| No consumption/1-2 days perweek |  |  |  |  | 1.00(ref) |
| 3-4 days per week | 0.066 | 0.105 | 0.396 | .529 | 1.07(0.87~1.31) |
| 5-7 days per week | 0.282 | 0.182 | 2.399 | .121 | 1.33(0.93~1.90) |
| **Frequency of salted fish and cured meats intake** |  |  |  |  |  |
| No consumption/1-2 days per week |  |  |  |  | 1.00(ref) |
| 3-4 days per week | 0.145 | 0.111 | 1.708 | .191 | 1.16(0.93~1.44) |
| 5-7days per week | 0.092 | 0.223 | 0.171 | .679 | 1.10(0.71~1.70) |
| **WBC(10^9^/L)** | 0.002 | 0.001 | 8.500 | **.003** | 1.00(1.00~1.00) |
| **PLT(10^9^/L)** | 0.000 | 0.000 | 0.955 | .328 | 1.00(1.00~1.00) |
| **HGB(g/L)** | -0.009 | 0.002 | 13.685 | **<.001** | 0.99(0.99~1.00) |
| **D-dimer(mg/L)** | 0.030 | 0.010 | 8.778 | **.003** | 1.03(1.01~1.05) |
| **CD4+** | -0.000 | 0.000 | 3.531 | .060 | 1.00(1.00~1.00) |
| **CD8+** | -0.000 | 0.000 | 2.365 | .124 | 1.00(1.00~1.00) |
| **CD3+** | -0.000 | 0.000 | 3.530 | .060 | 1.00(1.00~1.00) |
| **CD4+/CD8+** | -0.038 | 0.077 | 0.242 | .623 | 0.96(0.83~1.12) |
| **CRP(mg/L)** | 0.003 | 0.001 | 7.052 | **.007** | 1.00(1.00~1.01) |
| **GLU(mmol/L)** | 0.000 | 0.002 | 0.091 | .763 | 1.00(1.00~1.00) |
| **UA(umol/L)** | -0.000 | 0.000 | 0.039 | .843 | 1.00(1.00~1.00) |
| **ESR(mm/h)** | 0.004 | 0.006 | 0.483 | .487 | 1.00(0.99~1.02) |
| **CEA(ng/ml)** | 0.000 | 0.000 | 7.123 | **.007** | 1.00(1.00~1.00) |
| **CA12-5(U/ml)** | 0.000 | 0.000 | 12.906 | **<.001** | 1.00(1.00~1.00) |
| **CA19-9(U/ml)** | 0.000 | 0.000 | 6.431 | **.011** | 1.00(1.00~1.00) |
| **NSE(ng/ml)** | 0.001 | 0.000 | 2.817 | .093 | 1.00(1.00~1.00) |
| **CYFRA21-1(ng/ml)** | 0.002 | 0.001 | 8.747 | **.003** | 1.00(1.00~1.00) |
| **Location of lung cancer** |  |  |  |  |  |
| Both lungs |  |  |  |  | 1.00(ref) |
| Right lung | -0.139 | 1.003 | 0.019 | .890 | 0.87(0.12~6.21) |
| Left lung | -0.172 | 1.003 | 0.029 | .864 | 0.84(0.12~6.02) |
| **Histology** |  |  |  |  |  |
| SCLC/Other |  |  |  |  | 1.00(ref) |
| LUAD | -0.143 | 0.306 | 0.219 | .640 | 0.87(0.48~1.58) |
| LUSC | -0.150 | 0.318 | 0.224 | .636 | 0.86(0.46~1.60) |
| SCLC/Other | 0.193 | 0.330 | 0.344 | .558 | 1.21(0.64~2.32) |
| **Tumor stage** |  |  |  |  |  |
| T1+T2 |  |  |  |  | 1.00(ref) |
| T3+T4 | 0.268 | 0.093 | 8.207 | **.004** | 1.31(1.09~1.57) |
| **Nodal stage** |  |  |  |  |  |
| N1 |  |  |  |  | 1.00(ref) |
| N2 | 0.678 | 0.251 | 7.296 | **.006** | 1.97(1.20~3.22) |
| N3 | 0.909 | 0.196 | 21.615 | **<.001** | 2.48(1.69~3.64) |
| N4 | 1.076 | 0.196 | 30.264 | **<.001** | 2.93(2.00~4.31) |
| **Metastasis stage** |  |  |  |  |  |
| M1 |  |  |  |  | 1.00(ref) |
| M2 | 0.868 | 0.140 | 38.343 | **<.001** | 2.38(1.81~3.14) |
| **Stage** |  |  |  |  |  |
| StageI+StageII |  |  |  |  | 1.00(ref) |
| StageIII+StageIV | 1.086 | 0.234 | 21.537 | **<.001** | 2.96(1.87~4.69) |
| **TTF-1(+)** |  |  |  |  |  |
| No |  |  |  |  | 1.00(ref) |
| Yes | -0.013 | 0.089 | 0.020 | .886 | 0.99(0.83~1.17) |
| **CD56(+)** |  |  |  |  |  |
| No |  |  |  |  | 1.00(ref) |
| Yes | 0.385 | 0.161 | 5.719 | **.016** | 1.47(1.07~2.02) |
| **Ki67(+)** |  |  |  |  |  |
| No |  |  |  |  | 1.00(ref) |
| Yes | 0.012 | 0.089 | 0.018 | .894 | 1.01(0.85~1.20) |
| **Positive co-expression of CD56 and Ki67** |  |  |  |  |  |
| No |  |  |  |  | 1.00(ref) |
| Yes | 0.453 | 0.161 | 7.905 | **.004** | 1.57(1.15~2.16) |
| **ZPS GROUP A** |  |  |  |  |  |
| No |  |  |  |  | 1.00(ref) |
| Yes | -0.002 | 0.090 | 0.000 | .987 | 1.00(0.84~1.19) |
| **ZPS GROUP B** |  |  |  |  |  |
| No |  |  |  |  | 1.00(ref) |
| Yes | 0.586 | 0.219 | 7.144 | **.007** | 1.80(1.17~2.76) |
| **KPS GROUP A** |  |  |  |  |  |
| No |  |  |  |  | 1.00(ref) |
| Yes | 0.632 | 0.254 | 6.180 | **.012** | 1.88(1.14~3.10) |
| **KPS GROUP B** |  |  |  |  |  |
| No |  |  |  |  | 1.00(ref) |
| Yes | 0.367 | 0.124 | 8.724 | **.003** | 1.44(1.13~1.84) |
| **KPS GROUP C** |  |  |  |  |  |
| No |  |  |  |  | 1.00(ref) |
| Yes | -0.189 | 0.102 | 3.439 | .064 | 0.83(0.68~1.01) |
| **MDASI-LC GROUP A** |  |  |  |  |  |
| No |  |  |  |  | 1.00(ref) |
| Yes | -0.115 | 0.103 | 1.257 | .262 | 0.89(0.73~1.09) |
| **MDASI-LC GROUP B** |  |  |  |  |  |
| No |  |  |  |  | 1.00(ref) |
| Yes | 0.316 | 0.135 | 5.523 | **.018** | 1.37(1.05~1.79) |
| **MDASI-LC GROUP C** |  |  |  |  |  |
| No |  |  |  |  | 1.00(ref) |
| Yes | 0.183 | 0.183 | 1.000 | .317 | 1.20(0.84~1.72) |

Features with a p-value less than 0.05 (p < 0.05) are highlighted as statistically significant.

Abbreviations: CI, Confidence interval; BMI, Body Mass Index; WBC, White Blood Cell; PLT, Platelet Count; HGB, Hemoglobin; CD4+, CD4-Positive T-Lymphocytes; CD8+,CD8-Positive T-Lymphocytes; CD3+,CD3-Positive T-Lymphocytes; CD4+/CD8+, Ratio of CD4-Positive to CD8-Positive T-Lymphocytes; CRP,C-Reactive Protein; GLU, Glucose; UA, Uric Acid; ESR, Erythrocyte Sedimentation Rate; CEA, Carcinoembryonic Antigen; CA12-5,Cancer Antigen 125, CA19-9,Cancer Antigen 19-9; NSE, Neuron-Specific Enolase; CYFRA21-1,Cytokeratin-1 Fragment; LUAD, Lung Adenocarcinoma; LUSC, Lung Squamous Cell Carcinoma; SCLC, Small Cell Lung Cancer; MDASI-LC GROUP, Trajectory groups derived from the MDASI-LC scale through GBTM; KPS GROUP, Trajectory groups derived from the KPS scale through GBTM; ZPS GROUP, Trajectory groups derived from the ZPS scale through GBTM.

**Table S9.** The Baseline characteristics between training and testing dataset.

| **Variable** | **Dataset(N = 1,278)** | | **Statistic** | **P-value** |
| --- | --- | --- | --- | --- |
|  | **Training set**  **(N = 895)**  **n%** | **Testing set**  **(N = 383)**  **n%** |  |  |
| **Sex** |  |  |  |  |
| Male | 660(73.7%) | 286(74.7%) | *χ^2^*=0.12 | .775 |
| Female | 235(26.3%) | 97(25.3%) |  |  |
| **Age[*M*(*P*_25_,*P*_75_)]** | 62(54.0,68.0) | 61(55.0,67.0) |  | .939 ^a^ |
| **BMI[*M*(*P*_25_,*P*_75_)]** | 20.77(18.9,22.86) | 20.56(18.3,22.67) |  | .084 ^a^ |
| **Education** |  |  |  |  |
| Never attended school/Primary school | 516(57.7%) | 227(59.3%) | *χ^2^*=0.39 | .814 |
| Junior High School/High School | 344(38.4%) | 143(37.3%) |  |  |
| University and above | 35(3.9%) | 13(3.4%) |  |  |
| **Respiratory Disease History** |  |  |  |  |
| No | 764(85.4%) | 324(84.6%) | *χ^2^*=0.12 | .746 |
| Yes | 131(14.6%) | 59(15.4%) |  |  |
| **Smoking Status** |  |  |  |  |
| Never smoker | 360(40.2%) | 142(37.1%) | *χ^2^*=2.88 | .232 |
| Former smoker | 479(53.5%) | 223(58.2%) |  |  |
| Current smoker | 56(6.3%) | 18(4.7%) |  |  |
| **Second-hand smoking(SHS)** |  |  |  |  |
| No | 603(67.4%) | 262(68.4%) | *χ^2^*=0.13 | .751 |
| Yes | 292(32.6%) | 121(31.6%) |  |  |
| **Availability of an Exhaust Fan** |  |  |  |  |
| No | 31(3.5%) | 19(5%) | *χ^2^*=1.60 | .212 |
| Yes | 864(96.5%) | 364(95%) |  |  |
| **Frequency of fruit and vegetable intake** |  |  |  |  |
| No consumption/1-2 days per week | 72(8%) | 38(9.9%) | *χ^2^*=2.41 | .315 |
| 3-4 days per week | 571(63.8%) | 228(59.5%) |  |  |
| 5-7 days per week | 252(28.2%) | 117(30.5%) |  |  |
| **WBC(10^9^/L)[*M*(*P*_25_,*P*_75_)]** | 8.25(6.6,10.6) | 8.48(6.6,10.9) |  | .391 ^a^ |
| **PLT(10^9^/L)[*M*(*P*_25_,*P*_75_)]** | 288(227.0,362.0) | 297(227.5,366.0) |  | .264 ^a^ |
| **HGB(g/L)[*M*(*P*_25_,*P*_75_)]** | 123(110.0,134.0) | 124(112.0,136.5) |  | .145 ^a^ |
| **D-dimer(mg/L)[*M*(*P*_25_,*P*_75_)]** | 0.75(0.36,2.16) | 0.81(0.43,2.08) |  | .325 ^a^ |
| **CRP(mg/L)[*M*(*P*_25_,*P*_75_)]** | 14(2.23,41.29) | 17.31(2.05,46.11) |  | .711 ^a^ |
| **CEA(ng/ml)[*M*(*P*_25_,*P*_75_)]** | 6.21(2.79,30.09) | 6.32(2.68,34.48) |  | .887 ^a^ |
| **CA12-5(U/ml)[*M*(*P*_25_,*P*_75_)]** | 40.74(16.2,121.8) | 45.25(16.5,131.6) |  | .518 ^a^ |
| **CA19-9(U/ml)[*M*(*P*_25_,*P*_75_)]** | 13.84(6.31,31.9) | 14.29(7.19,30.3) |  | .575 ^a^ |
| **NSE(ng/ml)[*M*(*P*_25_,*P*_75_)]** | 14.99(11.4,23.6) | 15.42(11.3,25.8) |  | .710 ^a^ |
| **CYFRA21-1(ng/ml)[*M*(*P*_25_,*P*_75_)]** | 5.9(3.12,13.7) | 5.89(3.02,13.7) |  | .868 ^a^ |
| **Location of lung cancer** |  |  |  |  |
| Both lungs | 3(0.3%) | 2(0.5%) | *χ^2^*=1.53 | .497 ^a^ |
| Right lung | 520(58.1%) | 209(54.6%) |  |  |
| Left lung | 372(41.6%) | 172(44.9%) |  |  |
| **Histology** |  |  |  |  |
| LUAD | 609(68%) | 270(70.5%) | *χ^2^*=4.35 | .214 |
| LUSC | 176(19.7%) | 72(18.8%) |  |  |
| SCLC | 95(10.6%) | 30(7.8%) |  |  |
| Others | 15(1.7%) | 11(2.9%) |  |  |
| **Tumor stage** |  |  |  |  |
| T1 + T2 | 322(36%) | 139(36.3%) | *χ^2^*=0.01 | .948 |
| T3 + T4 | 573(64%) | 244(63.7%) |  |  |
| **Nodal stage** |  |  |  |  |
| N1 | 92(10.3%) | 39(10.2%) | *χ^2^*=0.77 | .853 |
| N2 | 70(7.8%) | 34(8.9%) |  |  |
| N3 | 384(42.9%) | 156(40.7%) |  |  |
| N4 | 349(39%) | 154(40.2%) |  |  |
| **Metastasis stage** |  |  |  |  |
| M1 | 176(19.7%) | 79(20.6%) | *χ^2^*=0.16 | .690 |
| M2 | 719(80.3%) | 304(79.4%) |  |  |
| **Stage** |  |  |  |  |
| Stage I-II | 66(7.4%) | 27(7%) | *χ^2^*=0.04 | .915 |
| Stage III-IV | 829(92.6%) | 356(93%) |  |  |

^a^ using the Mann-Whitney U test.

| 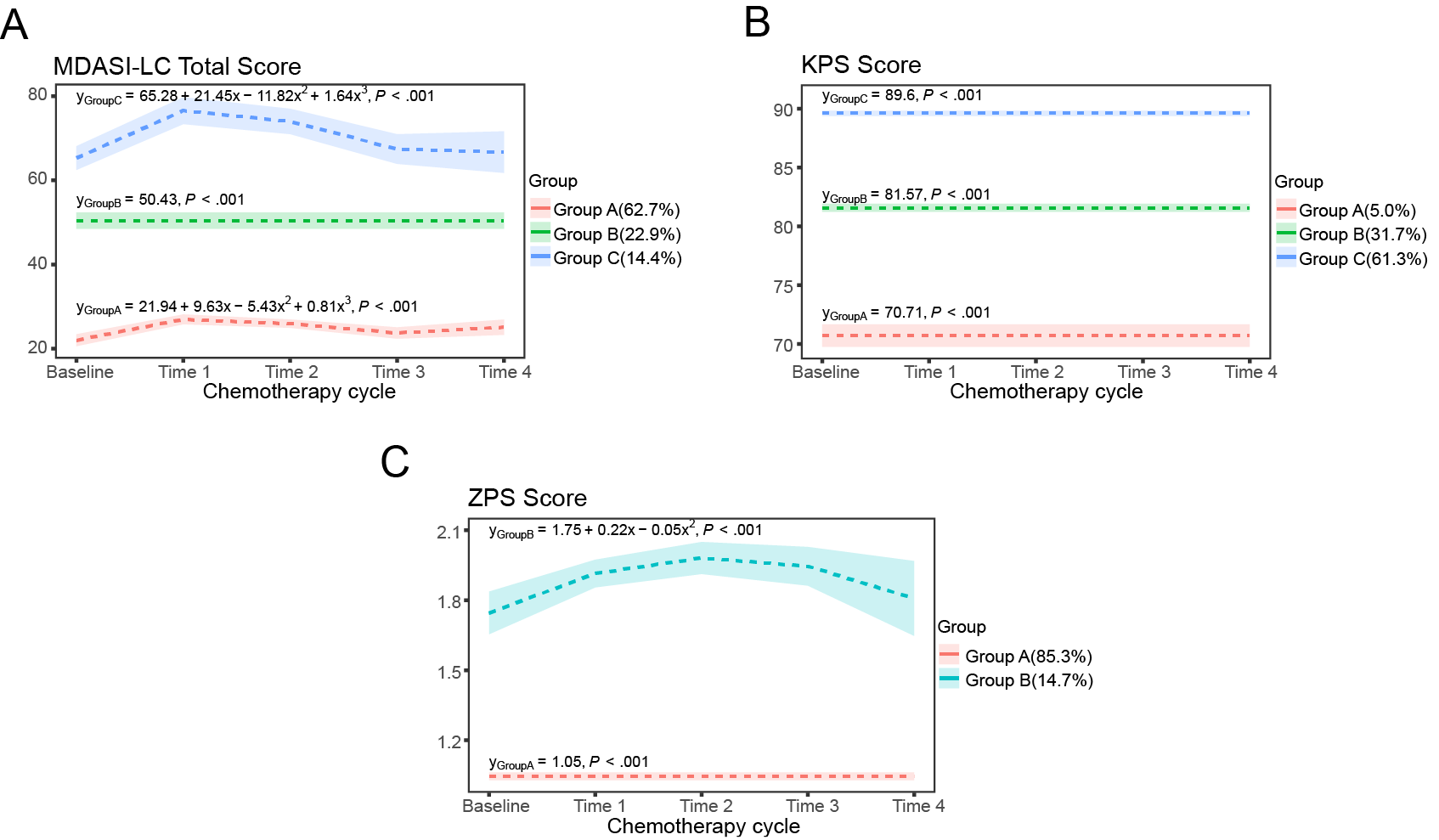 |
| --- |
| **Figure S1. PROs Trajectories After Chemotherapy in Lung Cancer.** Trajectories of symptom burden in lung cancer patients undergoing chemotherapy as assessed by patient-reported outcome (PRO). **(A)** to **(C)** represent the trajectory groups curve equations for the MD Anderson symptom inventory for lung cancer (MDASI-LC), the Karnofsky performance status (KPS), and the Zubrod score (ZPS) scales, respectively. The lines represent the estimated average scores of the scales along with their 95% confidence intervals (CIs). |

| 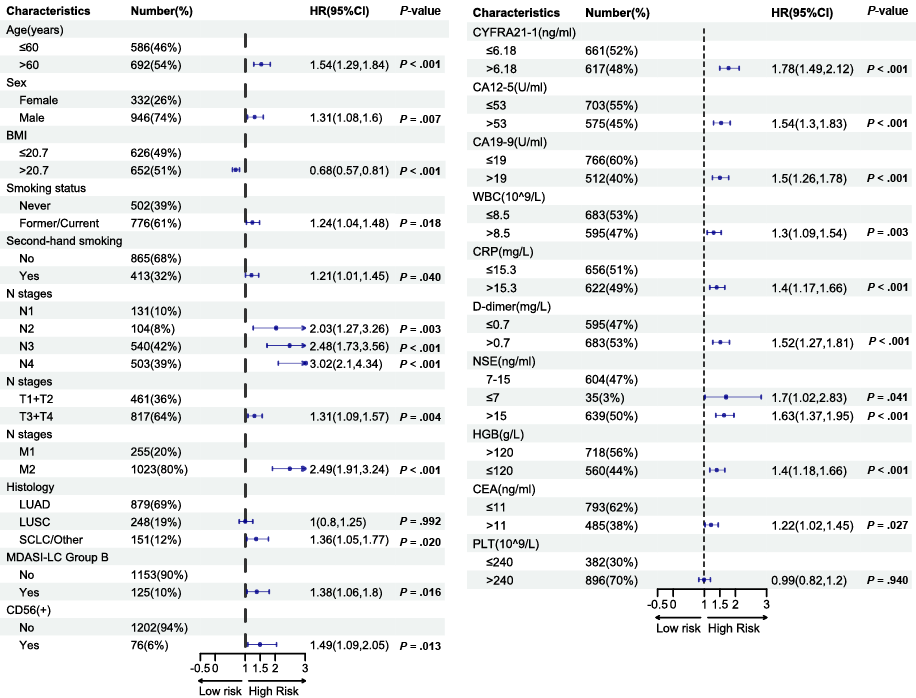 |
| --- |
| **Figure S2. Subgroup analysis validation after identifying key values through feature attribution analysis.**  Adjusted for gender, age, and smoking. Except for PLT, all characteristics contributed significantly to the prediction of mortality risk (*P*<.05) |
